# Silicon Application for the Modulation of Rhizosphere Soil Bacterial Community Structures and Metabolite Profiles in Peanut under *Ralstonia solanacearum* Inoculation

**DOI:** 10.3390/ijms24043268

**Published:** 2023-02-07

**Authors:** Quanqing Deng, Hao Liu, Qing Lu, Sunil S. Gangurde, Puxuan Du, Haifen Li, Shaoxiong Li, Haiyan Liu, Runfeng Wang, Lu Huang, Ronghua Chen, Chenggen Fan, Xuanqiang Liang, Xiaoping Chen, Yanbin Hong

**Affiliations:** 1 Guangdong Provincial Key Laboratory of Crop Genetic Improvement, South China Peanut Sub-Center of National Center of Oilseed Crops Improvement, Crops Research Institute, Guangdong Academy of Agricultural Sciences, Guangzhou 510640, China; 2 Department of Plant Pathology, University of Georgia, Tifton, GA 30602, USA; 3 Institute of Agricultural Sciences in Ganzhou, Ganzhou 341000, China

**Keywords:** metabolomics, peanut, silicon, peanut bacterial wilt, *Ralstonia solanacearum*, soil bacterial community

## Abstract

Silicon (Si) has been shown to promote peanut growth and yield, but whether Si can enhance the resistance against peanut bacterial wilt (PBW) caused by *Ralstonia solanacearum*, identified as a soil-borne pathogen, is still unclear. A question regarding whether Si enhances the resistance of PBW is still unclear. Here, an in vitro *R. solanacearum* inoculation experiment was conducted to study the effects of Si application on the disease severity and phenotype of peanuts, as well as the microbial ecology of the rhizosphere. Results revealed that Si treatment significantly reduced the disease rate, with a decrement PBW severity of 37.50% as compared to non-Si treatment. The soil available Si (ASi) significantly increased by 13.62–44.87%, and catalase activity improved by 3.01–3.10%, which displayed obvious discrimination between non-Si and Si treatments. Furthermore, the rhizosphere soil bacterial community structures and metabolite profiles dramatically changed under Si treatment. Three significantly changed bacterial taxa were observed, which showed significant abundance under Si treatment, whereas the genus *Ralstonia* genus was significantly suppressed by Si. Similarly, nine differential metabolites were identified to involve into unsaturated fatty acids via a biosynthesis pathway. Significant correlations were also displayed between soil physiochemical properties and enzymes, the bacterial community, and the differential metabolites by pairwise comparisons. Overall, this study reports that Si application mediated the evolution of soil physicochemical properties, the bacterial community, and metabolite profiles in the soil rhizosphere, which significantly affects the colonization of the *Ralstonia* genus and provides a new theoretical basis for Si application in PBW prevention.

## 1. Introduction

Cultivated peanut (*Arachis hypogaea* L.) is one of the most important leguminous oilseeds and food crops used for high-quality eatable oil and protein [1]. To date, ~54 million tons of peanuts are produced annually around the world [2,3], but peanut bacterial wilt (PBW) a soil-borne vascular disease caused by *Ralstonia solanacearum*, severely damages worldwide peanut production due to its widespread distribution and high pathogenicity [4,5,6]. Particularly, PBW causes ~100% yield loss under severe disease infestation [7]. Breeding disease-resistant peanut cultivars could be a cost-effective and sustainable approach to mitigate PBW [6]. However, there are a few systematic studies on PBW resistance which have reported that developing host resistance is challenging due to the allotetraploid genome of cultivated peanuts and polygenic quantitative traits associated with PBW resistance [8,9]. Therefore, it is crucial to develop strategies to control PBW and prevent yield loss in peanut fields.

Multiple strategies, including the management of agronomic practices [10], the use of chemical insecticides [11], and the application of soil amendment [12,13] had been implemented to prevent PBW infection in high-outbreak areas. Although the application of soil amendment with a balance between cost-effectiveness and environmental friendliness has been used to prevent bacterial wilt in other crops, such as tomatoes, tobacco, and potato [14,15,16], in peanut, there are also few reports on the mitigation of PBW. However, it is confirmed that the application of exogenous silicon (Si) via cost-efficient methods can improve plant resistance against soil-borne diseases [17,18]. Although Si is one of the most abundant elements on the Earth’s surface, there is always a deficiency of available Si in the soil because of acidic soil and rainy climates [19]. Recent studies reported that Si can induce broad-spectrum disease resistance by activating the plant immune system in Si-accumulating crops and Si-responsive crops [20,21]. The application of Si can significantly enhance the growth and yield of peanuts, especially in Si-deficient soil [22], though peanuts are reported as a Si-non-accumulator [23]. Si has been reported to improve resistance against bacterial wilt in tomato, and interestingly, tomato is also a Si-non-accumulating crop [21]. Numerous studies proved that Si could enhance the expression of disease resistance genes and proteins in tomato roots in response to *R. solanacearum* infection [21,24].

Most reports emphasizing the impact of Si on *R. solanacearum* mainly focused on mining transcriptome changes and screening markers associated with resistance in plants [4,8]. Although Si is considered to play an important role in soil conditioning and nutrient supply [25,26], the interactions between the environment and peanut-*R. solanacearum* are very poorly studied [27]. Plant roots absorb important mineral nutrients and release organic exudates, such as fatty acids [28], plant hormones [29], and antimicrobial compounds [30]. These organic exudates affect the interactions between plants and microbes; hence, the plant roots could help to structure the rhizosphere microbiome [31,32]. These organic compounds could be directly used as carbon and nitrogen sources by soil microorganisms [33]. The metabolites secreted by soil microbes also, in turn, affect plant root functions [34]. In addition, soil physiochemical properties, enzyme activities, and microbial communities are important for soil health [26]. Thereby, the root–soil–microbe interaction is certainly important for plants, e.g., in the protection of plants from soil-borne diseases/insects [35]. Further, studies have been conducted to elucidate this interaction to understand rhizosphere soil microbial community structures and metabolomics via high-throughput approaches in leguminous crops, such as soybean [36].

In the present study, we explored the effects of Si application on the peanut growth and disease rate of peanut bacterial wilt infections, and the physiochemical properties and enzyme activities of rhizosphere soil, the soil bacterial community, and soil metabolomics under *R. solanacearum* attack by watering a solution containing sodium silicate (3 mM). Based on our knowledge, this is the first systematic study used to explore the role of Si in order to enhance PBW resistance in the soil–peanut system and provide a new perspective to unravel the rhizosphere soil microbiome and metabolite induction during PBW resistance.

## 2. Results

### 2.1. PBW Disease Severity and Peanut Growth Responses

**Figure 1:**
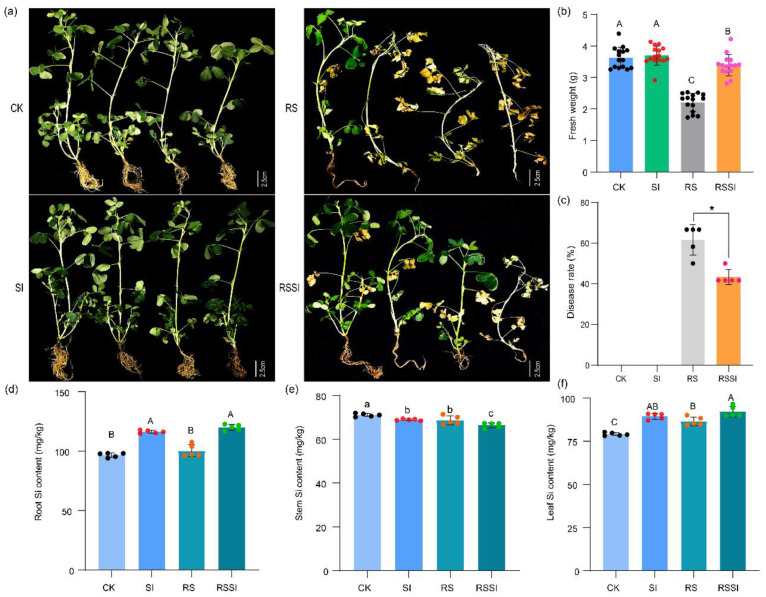
Fresh weight, disease rate, and Si contents of peanut plants at the 30th day after *R. solanacearum* inoculation. (**a**) Variation in the growth overview of peanut plants under CK, SI, RS, and RSSI treatments. (**b**) Fresh weight of peanut plants under CK, SI, RS, and RSSI treatments (capital letters indicate *p* < 0.01). (**c**) Disease rate of peanut plants under distinct treatments at the 30th day after inoculation (*, *p* < 0.05). (**d**–**f**) Si contents of the root, stem, and leaf of peanut plants under CK, SI, RS, and RSSI treatments (capitals and small letters indicate *p* < 0.01 and *p* < 0.05, respectively).

Interactions with the treatments showed that the fresh weight, the PBW disease rate, and Si contents of peanut plants were changed significantly (Figure 1). Under *R. solanacearum* infection, the fresh weight of plants under RSSI treatment was significantly higher (by 52.89%) as compared to RS treatment, while no significant difference was observed between the CK and SI treatments (Figure 1a,b). The symptoms of peanut bacterial wilt were first observed at 10 days post inoculation (dpi) in the plants of RS treatment, and the disease rate of RS and RSSI treatments was recorded at 30 dpi (Figure 1a,c). Compared to RS treatment, the disease severity on RSSI was significantly reduced, with a decrease in disease severity of 37.50%. Moreover, a significant difference in the Si contents of the root, stem, and leaf in peanut plants was observed between treatments, and most Si was retained in the roots (Figure 1d–f). Thereby, morphological observations indicated that Si application enhanced the resistance to PBW and provided basic information for further researching the disease-resistant mechanism in peanut plants.

### 2.2. Correlation between Disease Rate and the Other Investigated Parameters

All treatments had significant effects on soil physiochemical properties and enzymes (Table 1 and Table S1). The Si application had a significant effect on the increment of soil ASi content and the activity of S-CAT and S-ACP, while the available phosphorus (AP) and NO_3_^−^ showed a reverse behavior. Compared with the CK treatment, the addition of Si significantly increased (*p* < 0.01) the ASi at 13.62% (SI) and 44.87% (RSSI). Similarly, the S-CAT activity significantly improved (*p* < 0.05) under an SI of 3.10% and an RSSI of 3.01%. Conversely, the input of Si significantly decreased AP by 8.71% (SI) and 3.68% (RSSI) and NO_3_^−^ by 33.12% (SI) and 13.57% (RSSI) (Table S1, *p* < 0.01).

**Figure 2:**
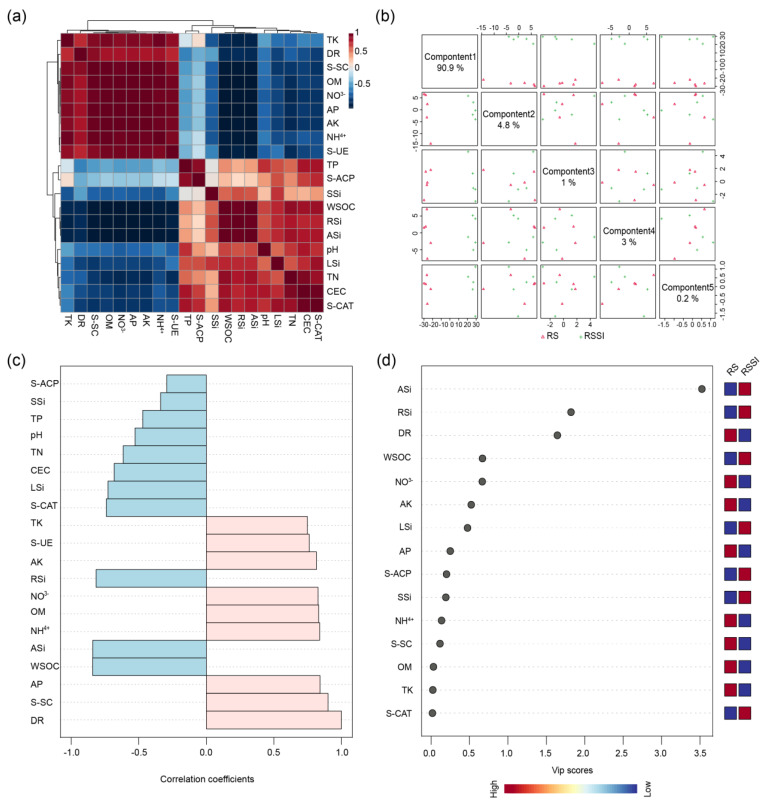
Impact of Si on rhizosphere soil physiochemical properties and enzymes under *R. solanacearum* inoculation. (**a**) Heat map for the DR and the investigated parameters. Red and blue grids represent positive and negative correlations, respectively. (**b**) Correlation of top 20 parameters with fresh weight. (**c**) Partial least squares-discriminant analysis (PLS-DA) of the 20 investigated parameters. (**d**) Variable importance in projection (VIP) to component 1 of the PLS-DA for RS and RSSI treatments.

The possible relationships between the disease rate (DR) and the other investigated parameters were further visualized with the help of a heat map (Figure 2a, Tables S2 and S3). This illustrated significantly negative correlations between the DR and several of the parameters such as WSOC, RSi, ASi, LSi, CEC, and S-CAT. However, the opposite parameters such as TK, S-SC, OM, NO_3_^−^, AP, AK, NH_4_^+^, and S-UE were positively correlated with DR (Figure 2a). Furthermore, the parameters that strongly correlated with the DR were shown to further reduce the redundant data (Figure 2b). We observed that the top 10 parameters, such as S-SC, AP, WSOC, ASi, NH_4_^+^, OM, NO_3_^−^, RSi, AK, and S-UE, were strongly correlated with the DR (Table S3). However, there are still too many data after screening, as shown in Figure 2b. Additionally, the PLS-DA of the investigated parameters indicated that component 1 (90.90%) accounted for most of the contributions under RS and RSSI treatments (Figure 2c). Moreover, ASi and RSi were the hub parameters based on the VIP value (Figure 2d). In fact, the further correlation analysis of the DR and the other parameters illustrated that Si application significantly affected rhizosphere soil properties which were remarkably associated with increased disease resistance.

### 2.3. Impact of Si on the Rhizosphere Soil Bacterial Community

**Figure 3:**
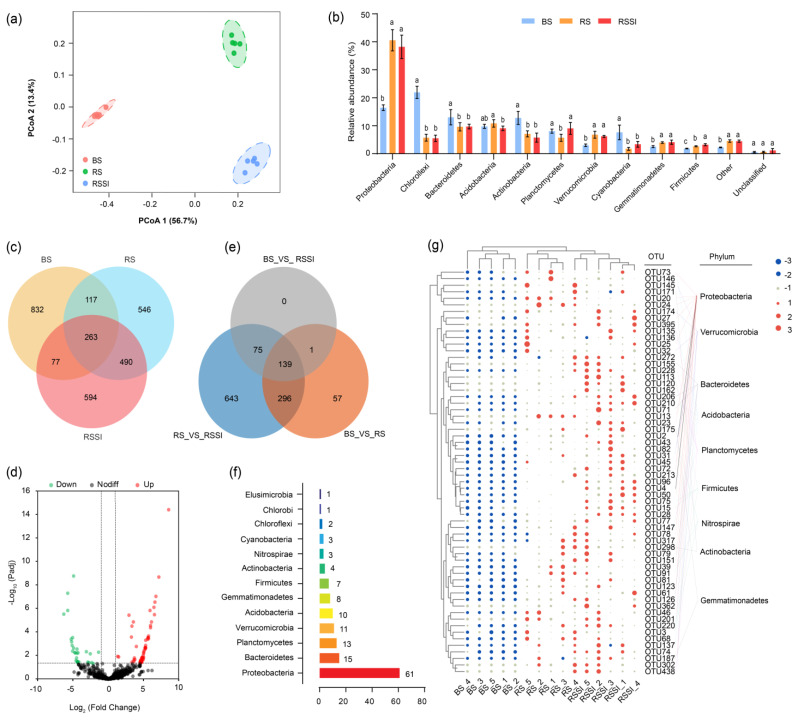
Impact of Si on the diversity of the rhizosphere soil bacterial community under *R. solanacearum* inoculation. (**a**) Beta diversity of the rhizosphere soil bacterial community. (**b**) Relative abundance of rhizosphere soil bacteria at the phylum level. (**c**,**e**) Venn diagrams illustrating the common and unique OTUs for each treatment and comparison group. (**d**) Differential relative abundance analysis showing up- and down-regulated OTUs under RS and RSSI treatments. (**f**) Bar chart presenting the number of common OTUs at the phylum level for the comparison groups. (**g**) Cluster heat map visualizing common OTUs among comparison groups (relative abundance > 0.1%).

In total, 230,063 filtered 16S sequences were obtained from 15 rhizosphere and basal soil samples (Table S4), whereas 6116–9111 bacterial sequences showed a 97% similarity level, and the number of bacterial operational taxonomic units (OTUs) ranged from 785 to 1450 in various soil samples (Table S4). For Alpha diversity analysis, the Chao, Shannon–Wiener, and Simpson indices indicated significant differences between the soil samples of the rhizosphere and basal soils (Table S5). For Beta diversity analysis, the clustering positions between distinct treatments were significantly separated (Figure 3a), which indicates that the community structure is particularly disturbed by Si treatments. Further analysis was accomplished to show differences in the soil bacterial beta diversity (R = 0.9991, *p* < 0.05) (Table 2). Additionally, the predominant bacterial phyla included *Proteobacteria*, *Chloroflexi*, *Bacteroidetes*, *Acidobacteria*, *Actinobacteria*, and *Planctomycetes* in BS, RS, and RSSI samples corresponding to the relative abundance of OTUs (Figure 3b). At the phylum level, *Planctomycetes* and *Firmicutes* showed significantly higher abundance levels in RSSI-treated soil as compared to RS-treated soil.

Moreover, the differential relative abundance of OTUs was also visualized on a Venn diagram and volcano plots to identify the core OTUs in Si application treatments (Figure 3c–e). Initially, the common and unique OTUs for each treatment were identified (Figure 3c). In combination RS_vs_RSSI treatment, 67 OTUs were upregulated, while 29 were downregulated with a reference of standard thresholds (fold change > 2.0, *p* < 0.05) (Figure 3d). Similarly, 139 OTUs shared between any two treatment groups were appraised and annotated based on their phyla taxon (Figure 3e,f, Table S6). Further, we identified 62 crucial OTUs with a relative abundance >5% and visualized their relative abundance patterns using a bubble heat map and by using clustering with phyla annotations under different treatments (Figure 3g, Table S7). In addition, 10 common OTUs were discovered in accordance with the crucial OTUs and the up- and down-regulated OTUs, which were composed of *Proteobacteria* (7), *Planctomycetes* (2), and *Firmicutes* (1) (Table S7). Particularly, at the genus level, a lower relative abundance of *Ralstonia* was observed in RSSI treatment as compared to RS treatment, whilst in BS, there was no abundance in the *Ralstonia* genus (Table S7). Further, core microbiome analysis was accomplished to anatomize the core rhizobacterial microbiome of each treatment, while OTU4 and OTU23 (*Proteobacteria*) were identified as core-OTUs (Table S8).

**Figure 4:**
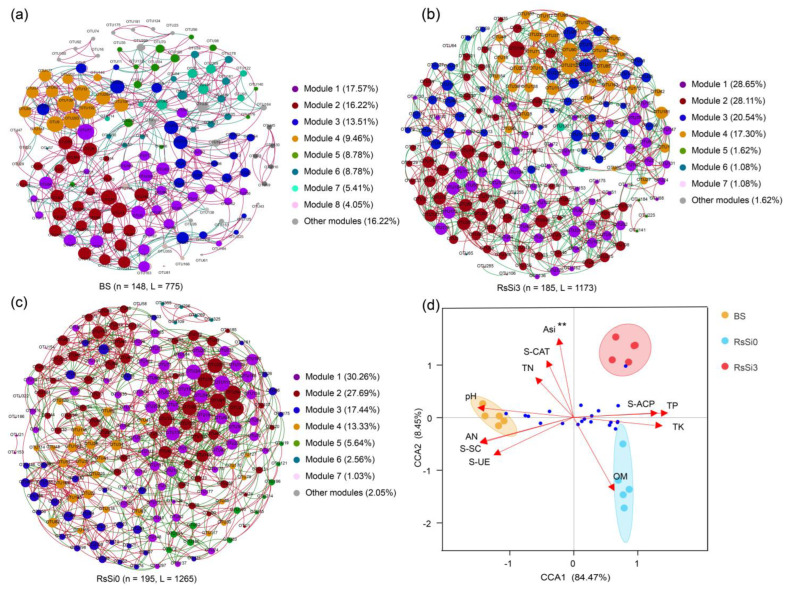
Impact of Si on the structures of the rhizosphere soil bacterial community under *R. solanacearum* inoculation. (**a**–**c**) Co-occurrence network module analysis of OTUs (relative abundance > 0.1%) under BS, RS, and RSSI treatments, respectively. Nodes were colored according to modularity type, and the connection of lines represents a strong correlation between bacterial OTUs (Spearman’s r > 0.8 and *p* < 0.05). A red edge indicates a positive interaction between two individual nodes, while a green edge indicates a negative interaction. (**d**) Canonical correspondence analysis (CCA) based on the bacterial community compositions at the genus level of BS, RS, and RSSI treatments samples. ** Significant at the 0.01 probability level.

In order to assess the rhizosphere soil bacterial community structures and to figure out the major driving factors under various Si treatments, co-occurrence network module analysis and canonical correspondence analysis (CCA) were performed (Figure 4). The rhizosphere bacterial interactions of all treatments were shown based on the network module, and the networks of the BS, RS, and RSSI treatments were each distributed into nine, eight, and eight modules, respectively (Figure 4a–c). In addition, in BS treatment, modules 1, 2, and 3 accounted for 17.57%, 16.22%, and 13.51% of the whole network, respectively (Figure 4a). In RS treatment, modules 1, 2, 3, and 4 accounted for 30.26%, 27.69%, 17.44%, and 13.33% of the whole network, respectively (Figure 4b). Meanwhile, in RSSI treatment, modules 1, 2, 3, and 4 accounted for 28.65%, 28.11%, 20.54%, and 17.30% of the whole network, respectively (Figure 4c). Therefore, the relationship between environmental factors (soil physiochemical properties and enzymes) and bacterial community structure was shown by the CCA model (Figure 4d). The CCA model showed that the CCA1 and CCA2 axes accounted for 85.01% and 6.94% of the total variations, whereas the first two axes together elucidated 91.95% of the taxonomic information at the genus level. Importantly, the ASi was highly correlated with the changes in bacterial community structures between treatments (Figure 4d).

### 2.4. Impact of Si on Rhizosphere Soil Metabolomics

Based on non-target metabolomics using LC-MS/MS, a total of 10,258 peaks in the positive ion mode (PIM) and 6433 peaks in negative ion mode (NIM) were generated (Table S9). Further annotation of the obtained data was used to identify metabolites by the secondary ion mass spectroscopy (MS2) analysis, and finally, a total of 425 and 298 of samples with identified peaks were determined in the PIM and NIM, respectively (Table S9).

**Figure 5:**
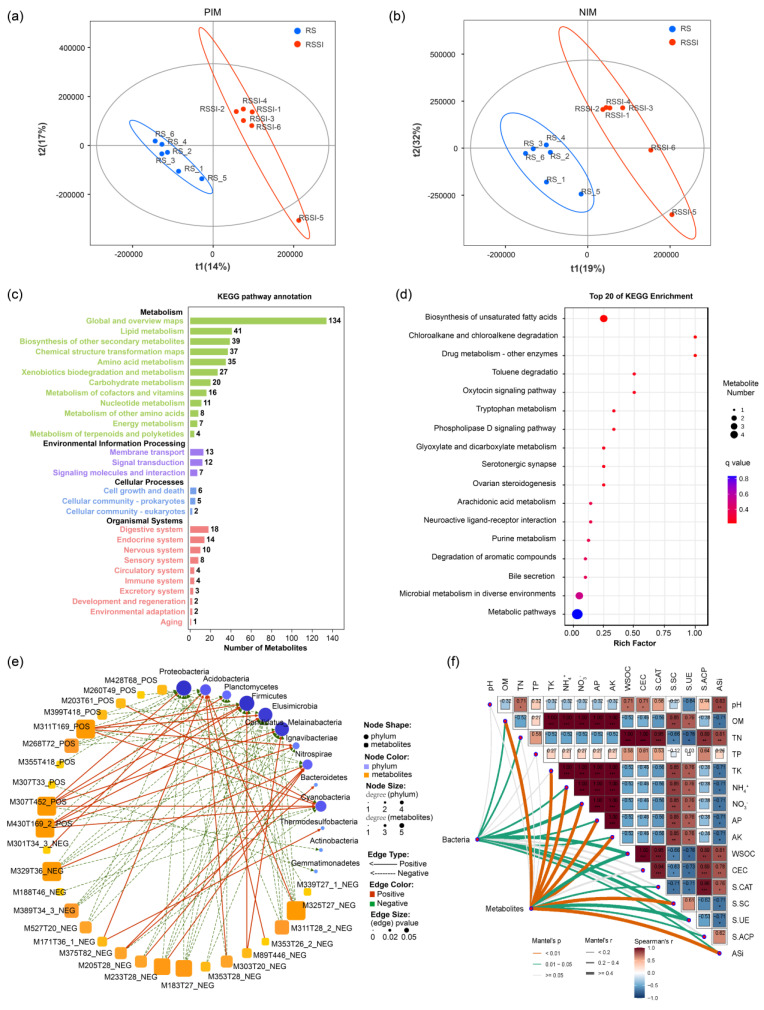
Impact of Si on rhizosphere soil metabolite profiles under *R. solanacearum* inoculation. (**a**,**b**) PLS-DA analyses showed the metabolite profiles of POS and NEG patterns in RS_vs_RSSI, respectively. (**c**) Annotation of the KEGG pathway enrichment analysis of total metabolites in all treatments. (**d**) Bubble map demonstrated the KEGG pathway enrichment analysis of differential metabolites in RS_vs_RSSI. (**e**) Co-occurrence network analysis of the Pearson correlation between differential metabolites of RS_vs_RSSI and the relative abundance of bacterial communities at the phylum level. (**f**) Pairwise comparisons of environmental factors, the bacterial community (genus level), and the differential metabolites with a color gradient indicating Spearman’s correlation coefficients. Rhizosphere soil bacterial and metabolite profiles were correlated with each environmental factor by Mantel tests. Spearman’s correlation coefficients were shown with a color gradient. Edge width and edge color denoted the Mantel’s r statistic and statistical significance levels, respectively.

Additionally, comparative metabolomics analysis was performed to establish the change in rhizosphere soil metabolic profiles, whilst PLS-DA model analysis showed significant differences between distinct treatments (Figure 5a,b and Figure S2). In total, 135, 164, and 116 annotated metabolites were obtained through in the PIM in BS_vs_RS, BS_vs_RSSI, and RS_vs_RSSI treatments, respectively (Table S10), whereas in the case of the NIM, 198, 196, and 114 annotated metabolites were obtained through NIM in BS_vs_RS, BS_vs_RSSI, and RS_vs_RSSI treatments, respectively (Table S11). Moreover, in the case of the PIM, 9, 19, and 12 of the differential metabolites (DEMs) in each treatment were distinguished. Meanwhile, in the NIM, 28, 12, and 19 of each treatment were identified in the various treatments based on VIP values >1 (Table 3, Figure S3). Importantly, six DEMs (M89T446_NEG, M430T169_2_POS, M307T452_POS, M311T169_POS, M203T61_POS, and M260T49_POS) displayed an up-regulation trend under RSSI treatment, indicating that RSSI treatment was clearly distinguished with the RS treatment (Table 3). Moreover, total metabolites and DEMs in rhizosphere soil were annotated with KEGG pathway enrichment analysis to show the top 20 active metabolic pathways under various treatments (Figure 5c,d and Figure S4). Particularly, the unsaturated fatty acids biosynthesis pathway was identified in the RS_vs_RSSI treatment (Figure 5d), and this pathway showed a high abundance of unsaturated fatty acids (e.g., M301T34_3_NEG eicosapentaenoic acid and M307T33_POS 11,14,17-eicosatrienoic acid) enriched in the RS treatment (Tables S10 and S11).

The data presented as a co-occurrence network module revealed the Pearson correlations of the DEMs with the rhizosphere soil bacterial phyla (Figure 5e, Table S12). At the phylum level, *Proteobacteria*, *Planctomycetes*, and *Firmicutes* were identified as the hub bacteria in the soil under *R. solanacearum* inoculation and Si treatment (Table S8). At the network module with the degree, *Proteobacteria* and *Firmicutes* were the key microorganisms that modulated the metabolomics in the rhizosphere soil (Figure 5d). Concomitantly, further analysis was conducted to determine the correlation between the DEMs of metabolomics, the bacterial community and environmental factors in the rhizosphere soil under *R solanacearum* inoculation (Figure 5f, Table S13). The DEMs and bacterial community significantly correlated with most of environmental factors (i.e., ASi, OM, NH_4_^+^, NH_3_^−^, AP, AK, WSOC, S-SC, and S-UE). Overall results illustrated that Si application had a higher impact on the rhizosphere soil bacterial community and metabolic profiles.

## 3. Discussion

In this study, we evaluated the impacts of Si treatment on the peanut growth response, rhizosphere soil physiochemical properties and enzymes, bacterial community structure, and metabolic profiles under *R. solanacearum* inoculation. Particularly, our results demonstrate highly influential environmental factors during plant-enhanced PBW resistance under Si application. In general, previous studies illustrated that Si could boost immunity and confer plant broad-spectrum resistance in Si-accumulating plants, such as rice, sugarcane, and wheat [37,38,39]. Recently, several studies showed that Si could improve the disease resistance of Si-non-accumulating plants against soil-borne pathogens [17,21,40]. Similarly, this study reported that Si application significantly reduced the disease rate of bacterial wilt in peanuts (Figure 1c). Moreover, all the above studies showed that Si mainly accumulated in the leaf and stem for non-soil-borne diseases [37,41], while tomato deposited Si in the root to protect against bacterial wilt [42]. Approximately, we also observed the rank of Si content in distinct parts of the peanut root, leaf, and stem (Figure 1d–f). Importantly, the root Si content of peanut was negatively correlated with the disease rate of peanut bacterial wilt, resulting in an obvious separation in the RS and RSSI treatments (Figure 2a–d).

The appropriate soil ecosystem functions are highly correlated with environmental factors such as soil physiochemical properties and enzymes, the soil microbial community structure, and soil metabolism [26,43]. Furthermore, it is also reported that Si can benefit the growth and health of peanuts by modulating environmental factors [26,44]. Our results demonstrate that the fresh weight of peanuts significantly improved under Si treatment and *R. solanacearum* infection; however, no remarkable increase was observed under Si utilization (Figure 1b). Simultaneously, the further measurement of rhizosphere soil properties revealed significant increases in the contents of ASi, WSOC, and CEC, while they remarkably decreased in the contents of OM, NH_4_^+^, NO_3_^−^, TK, AP, and AK (Table S1). Ma et al. (2021) illustrated that Si can probably promote the decomposition and absorption of nutrient elements in rice, resulting in a decrease in the nutrient elements’ content in the soil. Similarly, in our study, the applied Si solution only contained ultrapure water with sodium silicate, and Si could promote the uptake of nutrients by peanut plants in order to cause a decrease in rhizosphere soil nutrient contents, which was more prominent in the *R. solanacearum* infection treatments (Table S1). In addition, studies also confirmed that mineral Si (alkaline substance) enters the soil and carries more exchangeable cations at the surface [25,26]. In soil enzymes, we observed that S-CAT and S-ACP activities were significantly increased, but S-SC and S-UE activities were significantly decreased following the Si application (Table 1). We presumed that the enhanced enzyme activities might be due to the high WSOC content and its catalytic decomposition of hydrogen peroxide in the soil to reduce the toxic effects on plants [43,45]. In contrast, the S-SC and S-UE activities were correlated with the contents of OM and NH_4_^+^, which are associated with the carbohydrates’ metabolism in the soil [26,46].

The diversity and structure of soil microbial communities in the rhizosphere significantly affected soil quality and protected plants from microbial pathogens [32,47]. Generally, Si can influence the soil microbial community in the rhizosphere soil microenvironment in three main ways. The first is by directly modulating the microbial growth [17]; the second is by recognizing that Si can change soil microbial habitats by modifying soil physiochemical properties and enzymes; and the third is by recognizing that Si can induce the establishment of new advantaged microbial communities by altering rhizosphere soil metabolites [25,26,48,49]. Similarly, the findings of the present study suggested remarkable differences in the diversity and structure of the rhizosphere soil bacterial community in RS and RSSI treatments (Table S1, Figure 3). In particular, the alpha diversity index (e.g., Simpson) decreased and *Proteobacteria* were suppressed in the Si addiction treatment. These findings concurred with the findings proposed by Lin et al. (2020), which showed that *Proteobacteria* were suppressed in Si-treated soil as compared to the samples from the soil-borne diseased soil without Si treatment. Furthermore, the co-occurrence network module analysis and CCA separately demonstrated the significantly different community structure and ASi for the main driving factors in the distinct treatments. It is also reported that Si application is the key factor that affected the incidence of ginseng black spot and microbial community structures in soil [50]. Previous studies reported that the bacteria of the *Ralstonia* genus are dominant in wilt-diseased soil [40,51]. Notably, Si application caused a lower relative abundance of the *Ralstonia* genus in rhizosphere soil, which is the same genus as *R. solanacearum* (Table S7), which proves the role of Si in reducing the disease rate of peanut bacterial wilt.

Concomitantly, rhizosphere metabolites play an important role in diversifying the microbial community structure [43,49]. In the current investigation, the RS treatment revealed significantly higher levels of several DEMs compared to the Si application treatment, which are enriched in the biosynthesis pathways of unsaturated fatty acids. Previous studies also confirmed that the types and contents of unsaturated fatty acids in *R. solanacearum* are associated with their pathogenicity [52,53]. These results indirectly verify the high abundance of the *Ralstonia* genus in the soil bacterial community in the no-Si treatment (Table S7). Similarly, the interaction network analysis showed that most of the DEMs belonged to fatty acids, which are the core factors influencing the phyla abundance of *Proteobacteria* and *Firmicutes* (Table S12). Si can induce the root exudates of organic acids to affect the soil microbial community [17,54]. In this study, we observed a highly significant connection between soil physiochemical properties and enzymes, bacterial community, and the DEMs based on Si application under *R. solanacearum* infection. In summary, all the findings suggest that Si could induce the formation of new dominant and beneficial microorganisms in the rhizosphere of soil by changing the soil environment, including soil physiochemical properties and enzymes, microbial metabolites, and the microbial community.

## 4. Materials and Methods

### 4.1. Experimental Materials

The experimental basal soil (BS), collected from an experimental base (23°23′N, 113°26′E) in Guangdong Province, China, was acidic. A bacterial wilt susceptible peanut cultivar, Zhaonghua12, was used in the present study, and seeds were sterilized with 0.1% (*v*/*v*) bleach for 15 min, washed in sterile water, and sown on basal soil [55]. Seeds were grown in a glasshouse with natural light. A strain of *R. solanacearum* with a strongly aggressive defoliation was applied to the experiment of peanut inoculation [56]. TTC solid medium was used to revitalize the storage of glycerol (final concentration 25%) of *R. solanacearum*, stored in an ultra-cold storage freezer, and incubated at 28 °C for 48–72 h [4]. The typical pink colonies (Figure S1) with a white halo and an irregular fluidal shape were expandingly propagated by conical flasks containing LB liquid medium (10 g/L of tryptone, 10 g/L of NaCL, and 5 g/L of yeast extract) and incubated at 28 °C for 24 h and 200 r/min on a shaker.

### 4.2. Experimental Treatments and Phenotype Assays

The pot experiment was conducted in a randomized block design by using four Si fertilizer treatments including controls: (i) CK treatment—plants were irrigated by ultrapure water without soluble Si, and not inoculated with *R. solanacearum*; (ii) SI treatment—plants were irrigated with a 3 mM sodium silicate solution, but not inoculated with *R. solanacearum*; (iii) RS treatment—plants were irrigated with ultrapure water without soluble Si, but inoculated with *R. solanacearum*; (iv) RSSI treatment—plants were irrigated with a 3 mM sodium silicate solution and inoculated with *R. solanacearum* [48]. Each treatment was performed in quintuplicate with 12 plants in each replication. For RS treatment, sodium chloride was used to replenish sodium. The plants were watered every two days with 30 mL of the corresponding solution (pH 6.8). At the six-leaf stage, the peanut plants were inoculated with 20 mL of the *R. solanacearum* pathogen suspension culture of concentration (4 × 10^9^ CFU/mL) by infusing the roots in one pot [56]. The CK and SI treatments were inoculated with sterile water.

The PBW disease rate of each treatment was recorded when the symptoms of bacterial wilt were found for the first time until no new infected plants appeared (at 30 days). The PBW disease rate in each replication was calculated using the following formula: (PBW incidence (%) = diseased plants/total number of plants) × 100%. Five biological replicates were performed with 12 plants per replicate, and 60 peanut plants (mean total) were inoculated in each experiment. After statistically analyzing the data on the disease ratings of bacterial wilt, the fresh weight of each plant was measured. In order to estimate the Si from the peanut plants, the roots, stems, and leaves were sampled from five plants individually per pot and dried at 110 °C for 30 min and then at 80 °C to obtain a constant mass of dry matter for each sample. A high-speed grinder was used to grind the dried samples, which was then passed through a 100-mesh sieve. Finally, the powdered sample for each replicate was used to estimate the total Si content via the high-temperature alkaline melting method [37].

### 4.3. Rhizosphere Soil Sampling and Measurement

The total rhizosphere soil of total plants of RS and RSSI plant treatments was removed at 30 dpi by shaking the root manually; meanwhile, the basal soil (BS, non-rhizosphere soil) was collected as the control [57]. Furthermore, the soil samples were mixed and used for three separate determinations of soil chemical properties, soil enzymes and soil microorganisms, and metabolite profiles. In general, the subsample of soil properties was air-dried to estimate the available soil Si (ASi), pH, organic matter (OM), total nitrogen (TN) content, total phosphorus (TP) content, total potassium (TK) content, ammonia (NH_4_^+^) content, nitrate (NO_3_^−^) content, available phosphorus (AP), available potassium (AK), water-soluble organic carbon (WSOC) and cation exchange capacity (CEC) [48]. Soil catalase (S-CAT), sucrase (S-SC), and urease (S-UE) and acid phosphatase (S-ACP) activities were determined using the methods of Abdul Rahman et al. [58]. Another subsample was frozen in an ultra-low temperature (−80 °C) freezer for soil DNA extraction and bacterial community analysis. In addition, the soil properties of the basal soil comprised pH 6.59 soil, 12.99 g kg^−1^ of OM, 0.85 g kg^−1^ of TN, 0.56 g kg^−1^ of TP, 8.34 g kg^−1^ of TK, 8.06 mg kg^−1^ of NH_4_^+^, 9.41 mg kg^−1^ of NO_3_^−^, 64.82 mg kg^−1^ of AP, 174.28 mg kg^−1^ of AK, 174.28 mg kg^−1^ of WSOC, 7.56 cmol kg^−1^ of CEC, and 99.45 mg kg^−1^ of ASi, and the enzyme activities of S-CAT, S-SC, S-UE, and S-ACP were 2.35 mL^−1^ g^−1^ 20 min, 7.56 mg^−1^ g^−1^ 24 h, 0.69 mg^−1^ g^−1^ 24 h, and 94.57 µg^−1^ g^−1^ 2 h, respectively.

### 4.4. PacBio Sequencing

The total soil DNA was extracted from each soil sample (0.5 g) using the HiPure Soil DNA Kits (D3142, Magen Biotechnology Co., Ltd., Guangzhou, China). The quality of the extracted DNA was determined by a NanoDrop 2000 microspectrophotometer (Thermo Fischer Scientific, Wilmington, DC, USA) and agarose gel electrophoresis, respectively. Moreover, the primers 27F (5′-AGRGTTTGATYNTGGCTCAG-3′) and 1492R (5′-TASGGHTACCTTGTTASGACTT-3′) were performed for full-length (V1 to V9) 16S rDNA gene amplification (PacBio sequencing) [59]. The amplification program was set as 1 cycle of 95 °C for 5 min, 30 cycles at 95 °C for 1 min, annealing at 60 °C for 1 min, primer extension at 72 °C for 1 min and a final extension at 72 °C for 7 min. The PCR reactions were conducted in triplicate by Q5^®^ high-fidelity DNA polymerase (M0491, New England Biolabs, Ipswich, MA, USA). Sequencing libraries were generated using SMRTbell TM Template Prep Kit (PacBio, Menlo Park, CA, USA), following the recommendations given by the manufacturer. The libraries were sequenced on the PacBio Sequel platform. The raw sequencing data were uploaded to the China National Center for Bioinformation under Bio-project accession CRA013815.

After PacBio 16S sequencing, the raw fastq files were assigned to samples based on their unique barcode and PacBio’s open-source software suite SMRT Link (version 7.0) was used to generate circular consensus sequencing (CCS) reads. The UPARSE (version 9.2.64) pipeline was used to cluster the retained clean reads into operational taxonomic units (OTUs) of ≥97% similarity [60]. Based on the representative OTU sequences, taxonomic classification and annotation were conducted by BLAST (version 2.6.0) on the RNA Database (www.ncbi.nlm.nih.gov) accessed on 1 September 2022 [61]. In brief, biological information analysis was carried out based on the OTUs and species abundance tables.

The “vegan” package in R V4.1.3 was used to perform principal co-ordinate analysis (PCoA), a subgroup significance test (analysis of similarity, ANOSIM), co-occurrence network module analysis and canonical correspondence analysis (CCA) [62]. The Venn and volcano plots were used for discriminating OTUs which significantly correlated with community separation among treatments. A cluster heat map was used to visualize unique OTUs and comparisons of groups were made by TBtools [63].

### 4.5. LC-MS/MS Based Soil Metabolomics

Defrosted rhizosphere soil samples at 4 °C were dissolved in pre-chilled methanol–acetonitrile–water solution (2:2:1, *v*/*v*), mixed on a vortex mixer and then sonicated at a low temperature for 30 min. The soil solutions were left to stand for 10 min at −20 °C and centrifuged at 14,000× *g* for 20 min at 4 °C. The supernatant was freeze-dried and the dried sample was re-dissolved in the 100 μL aqueous acetonitrile solution (acetonitrile: water = 1:1, *v*/*v*). All sample solutions were vortexed and centrifuged at 14,000× *g* for 15 min (4 °C), and the supernatant was used for mass spectrometry analysis. Each treatment was performed in six independent biological replicates.

The rhizosphere soil metabolites were analyzed by ultra-high performance liquid chromatography and mass spectrometry. All metabolites were analyzed by Agilent 1290 Infinity LC (liquid chromatography) (Agilent Technologies Inc., Santa Clara, CA, USA). The sample was placed in the auto sampler at 4 °C during the whole analysis. QC samples were inserted in the sample queue for monitoring and evaluating the stability of the system and the reliability of the experimental data. Furthermore, an AB Triple TOF 6600 mass spectrometer (AB SCIEX Inc., MA, USA) was used for the acquisition of the primary and secondary spectra of the samples.

The metabolites were visualized by partial least squares-discriminate analysis (PLS-DA) and orthogonal projection to latent structures-discriminant analysis (OPLS-DA) under positive ion mode (PIM) and negative ion mode (NIM) [43]. Moreover, a metabolite was identified as a differential metabolite (DEM if the variable importance for the projection (VIP) value of the OPLS-DA model was greater than 1 and the *p* values were less than 0.05 (two-tailed Student’s *t*-test) [64]. The KEGG database (www.kegg.jp) accessed on 6 September 2022 was used to annotate all metabolites and their metabolic pathway enrichment analysis was performed. Meanwhile, the bubble map was used to show the enrichment results of the top 20 metabolic pathways.

### 4.6. Statistical Analysis

Significant differences in physiological parameters (fresh weight, disease rate, Si content, and soil chemical properties and enzymes) between the different treatments were analyzed by one-way ANOVA using Statistix V8.0 (Analystical, Tallahassee, FL, USA) [43]. For the multivariate analysis of parameters, MetaboAnalyst software (www.metaboanalyst.ca) accessed on 10 October 2022 was used and the results were visualized using a heat map, pattern hunter and PLS-DA [65]. Moreover, the hub parameters were estimated by PLS-DA based on VIP values >1. The differences in parameters are illustrated using bar charts generated using GraphPad Prism version 8.0.0 for Windows (GraphPad Software, San Diego, CA, USA).

## 5. Conclusions

The study reports that Si application can significantly enhance the disease resistance against PBW and promote peanut growth to some extent. Similarly, in rhizosphere soil properties and enzyme activities, the improvement of ASi was remarkably associated with disease resistance, while Si notably increased the S-CAT activity, and S-SC and S-UE showed the opposite trend. Si application helped to improve the bacterial community structures in rhizosphere and metabolite profiles under *R. solanacearum* inoculation in peanut. The bacterial community structures and metabolite profiles were strongly correlated with the soil physiochemical properties and enzyme activities. Overall, the above results of this study provided a theoretical basis for Si application as a control measure for PBW prevention from the perspective of soil microbial ecology.

## Figures and Tables

**Table 1:** Responses of rhizosphere soil physiochemical properties and enzymes under CK, SI, RS, and RSSI treatments.

Treatment	S-CAT	S-SC	S-UE	S-ACP
	mL/g/20 min	mg/g/24 h	mg/g/24 h	µg/g/2 h
CK	2.33 ± 0.07 ab	4.23 ± 0.03 C	0.64 ± 0.01 A	134.88 ± 1.67 A
SI	2.26 ± 0.08 ab	4.70 ± 0.01 B	0.58 ± 0.01 B	138.30 ± 2.90 A
RS	2.19 ± 0.05 b	4.98 ± 0.06 A	0.61 ± 0.01 A	109.58 ± 3.33 B
RSSI	2.40 ± 0.02 a	3.61 ± 0.07 C	0.53 ± 0.01 C	111.95 ± 1.33 B

Note: Capital and small letters indicate *p* < 0.01 and *p* < 0.05, respectively. Same letters indicate no significant difference.

**Table 2:** Analysis of similarity (ANOSIM) analysis of rhizosphere soil bacterial community structure between treatments.

Groups	R_Value	*p*_Value	Sig
BS_vs_RSSI	1	0.014	*
BS_vs_RSSI	1	0.013	*
RS_vs_RSSI	0.864	0.007	**
BS_vs_RS_vs_RSSI	0.8293	0.001	**

Note: ** and * indicate *p* < 0.01 and *p* < 0.05, respectively.

**Table 3:** KEGG pathway classes of DEMs in RS_vs_RSSI treatment.

ID	Name	Class	Pathway	Fold
PIM				
M260T49_POS	Venlafaxine	Organooxygen compounds	Global and overview maps	1.42
M430T169_2_POS	Sorbitane monostearate	Fatty acid	Lipid metabolism	2.12
M307T33_POS	11,14,17-Eicosatrienoic acid	Fatty acid		0.68
M311T169_POS	Oleic acid ethyl ester	Fatty acid		2.24
M268T72_POS	Auramine o	Organic chloride salt	Biosynthesis of other secondary metabolites	0.68
M533T33_POS	Manumycin a	Organooxygen compound	Chemical structure transformation maps	0.68
M223T597_POS	Tetradecanedioic acid	Organooxygen compound		0.83
M399T418_POS	Ethiprole	Monocyclic heteroarene		0.64
M428T68_POS	Peimisine	Alkaloid		0.68
M307T452_POS	Perchloroterephthalic acid	Organooxygen compound	Carbohydrate metabolism	1.9
M355T418_POS	Carboplatin	Cyclobutanedicarboxylate	Metabolism of other amino acids	0.64
M203T61_POS	Cyclohexanone	Ketone	Metabolism of terpenoids and polyketides	1.68
NIM				
M353T26_2_NEG	Prostaglandin f2.alpha.	Fatty acid	Lipid metabolism	0.23
M618T34_NEG	N-palmitoyl-d-erythro-dihydroceramide-1-phosphate	Fatty acid		0.55
M375T82_NEG	(R)-butaprost	Fatty acid		0.63
M375T37_NEG	Hexaenoic acid	Fatty acid		0.62
M527T20_NEG	Pachymic acid	Fatty acid		0.18
M301T34_3_NEG	Eicosapentaenoic acid	Fatty acid		0.56
M389T34_3_NEG	Ilicicolin a	Phenolate	Chemical structure transformation maps	0.78
M339T27_1_NEG	Gly-His-Lys	Amino acids	Amino acid metabolism	0.72
M89T446_NEG	Oxalate	Amino acids, peptides, and analogues		1.4
M325T27_NEG	Hydroquinidine	Organonitrogen compound	Xenobiotics biodegradation and metabolism	0.78
M205T28_NEG	Hydroxybenzoic acid	Benzoic acids and derivatives		0.73
M171T36_1_NEG	p-Toluenesulfonic acid	Benzoic acids and derivatives		0.61
M329T36_NEG	Aurantio-obtusin	Anthraquinones		0.54
M311T28_2_NEG	Thymol-beta-d-glucoside	Carbohydrate derivative	Carbohydrate metabolism	0.83
M183T27_NEG	Thiouric acid	Purines and purine derivatives	Nucleotide metabolism	0.77
M353T28_NEG	Rauwolscine	Alkaloid	Energy metabolism	0.84
M188T46_NEG	Kynurenic acid	Quinoline carboxylic acids		0.69
M303T20_NEG	2,6-Dihydroxy-7-methoxy-1,1,4a-trimethyl-3,4,10,10a-tetrahydro-2h-phenanthren-9-one	Terpenoid	Metabolism of terpenoids and polyketides	0.11
M233T28_NEG	Valerenic acid	Terpenoid		0.7

## Data Availability

Data supporting the discovering of our work are available within the paper and its Supplementary Information files.

## Supplementary Materials

The following supporting information can be downloaded at: https://www.mdpi.com/article/10.3390/ijms24043268/s1.
