# Supplementary material for: Silicon Application for the Modulation of Rhizosphere Soil Bacterial Community Structures and Metabolite Profiles in Peanut under Ralstonia solanacearum Inoculation
Source: Int J Mol Sci. 2023 Feb 7;24(4):3268. doi: 10.3390/ijms24043268 (PMC9960962; doi:10.3390/ijms24043268)
Supplement: Supplementary file 1 [file ijms-24-03268-s001.zip › Supplementary Figures.pdf]

Title:

Silicon Application for the Modulation of Rhizosphere Soil Bacterial Community Structures and Metabolite Profiles in Peanut under Ralstonia solanacearum Inoculation

Author Name:

Quanqing Deng[1,#], Hao Liu[1,#], Qing Lu[1], Sunil S. Gangurde[2], Puxuan Du[1], Haifen Li[1], Shaoxiong Li[1], Haiyan Liu[1], Runfeng Wang[1], Lu Huang[1], Ronghua Chen[3], Chenggen Fan[3], Xuanqiang Liang[1], Xiaoping Chen[1,*], Yanbin Hong[1,*]

#These authors contribute equally to this work.

Affiliations:

[1]Guangdong Provincial Key Laboratory of Crop Genetic Improvement, South China Peanut Sub-Center of National Center of Oilseed Crops Improvement, Crops Research Institute, Guangdong Academy of Agricultural Sciences, Guangzhou, 510640, China

[2]Department of Plant Pathology, University of Georgia, Tifton, GA, 30602, USA

[3]Institute of Agricultural Sciences in Ganzhou, Ganzhou, 341000, China

Corresponding Author:

*Correspondence, email: hongyanbin@gdaas.cn, chenxiaoping@gdaas.cn

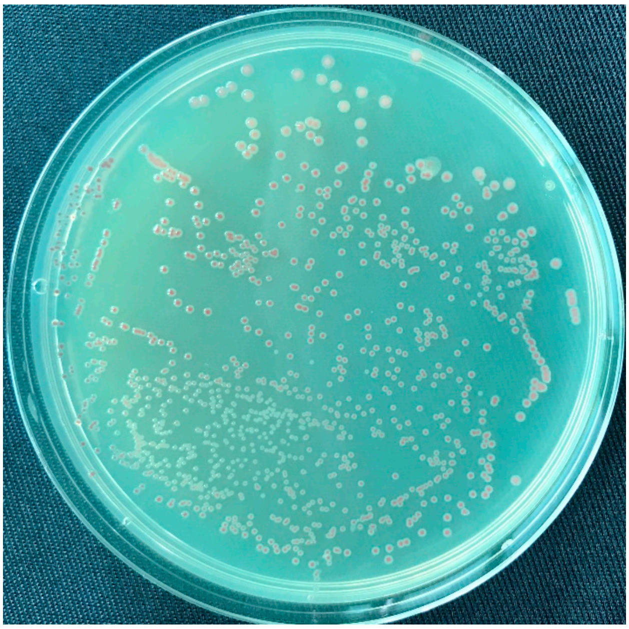

Figure S1. *Ralstonia solanacearum* incubated at TTC solid medium

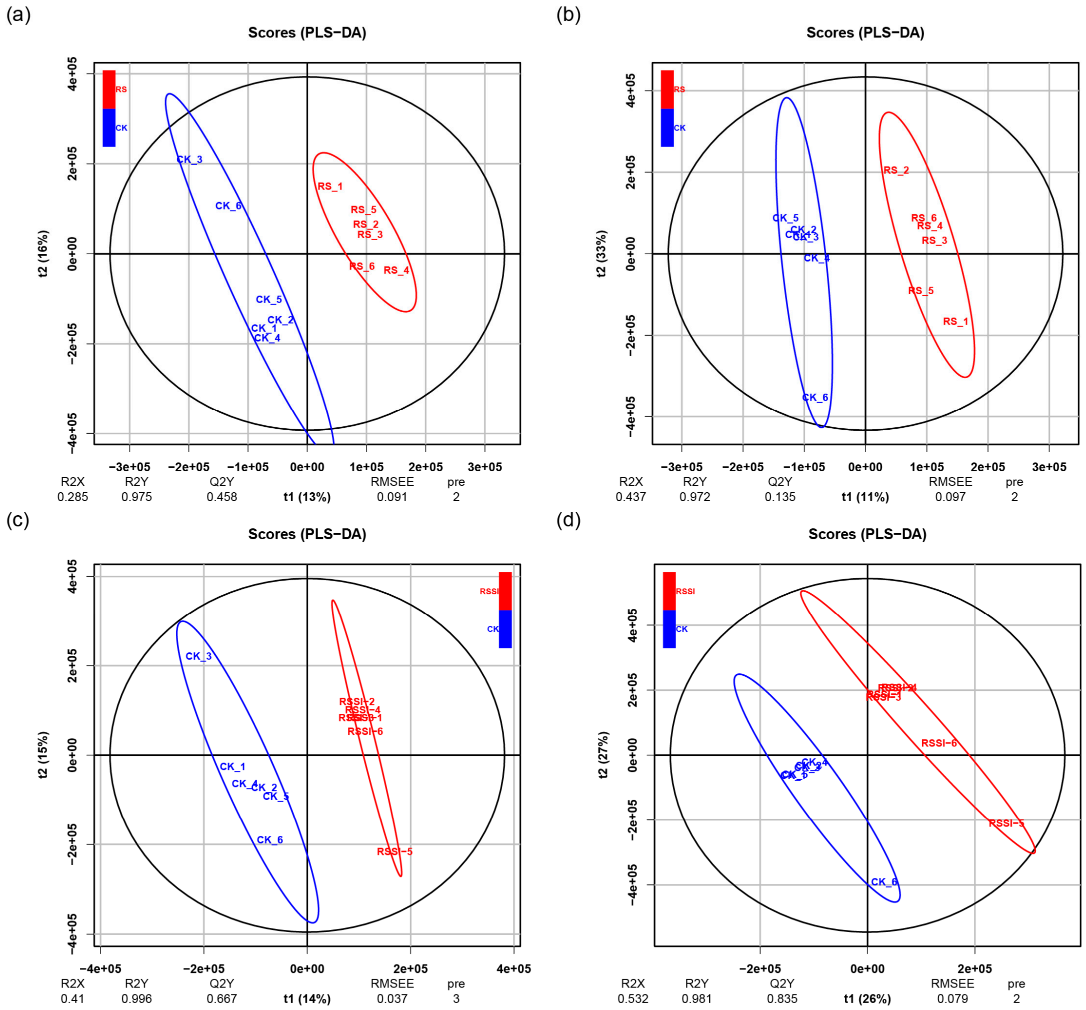

Figure S2. PLS−DA analysis showed the metabolite profiles of POS and NEG patterns in BS_vs_RS (a) and (b) and BS_vs_RSSI (c) and (d), respectively.

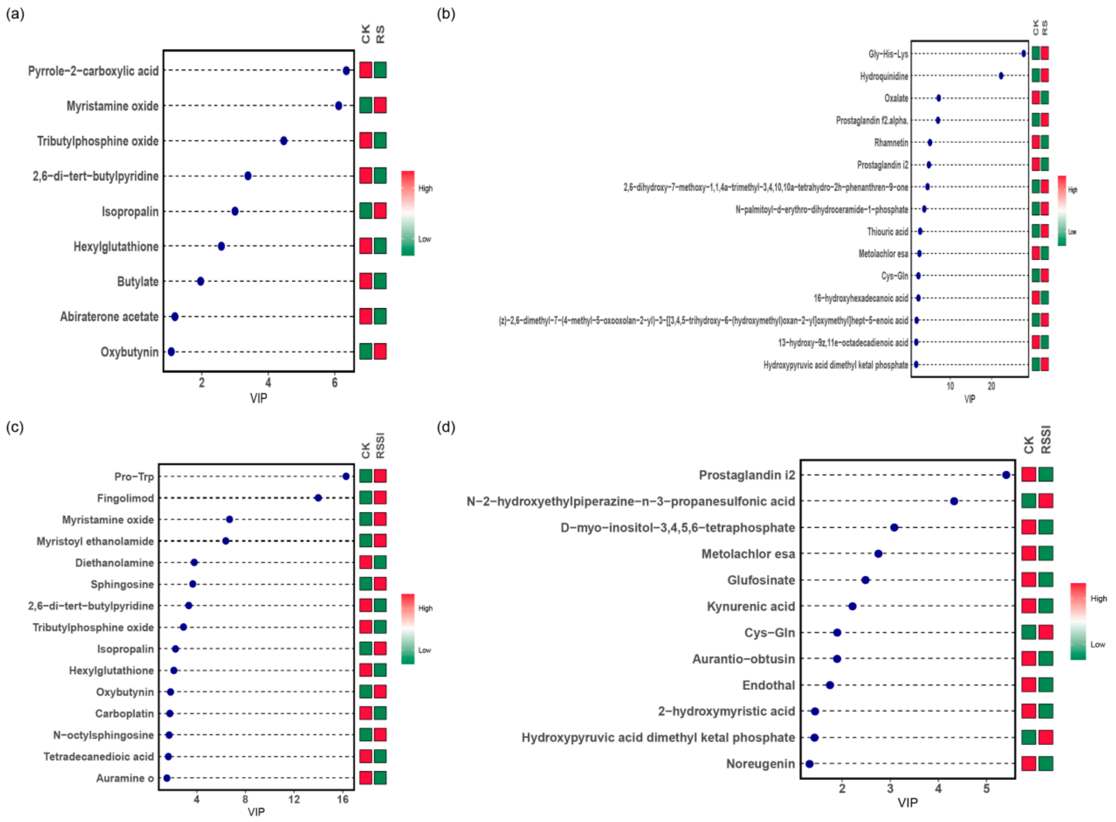

Figure S3. The OPLS−DA analysis showed the differential metabolites in the top 15 of VIP values of the POS and NEG patterns in BS_vs_RS (a and b) and BS_vs_RSSI (c and d), respectively.

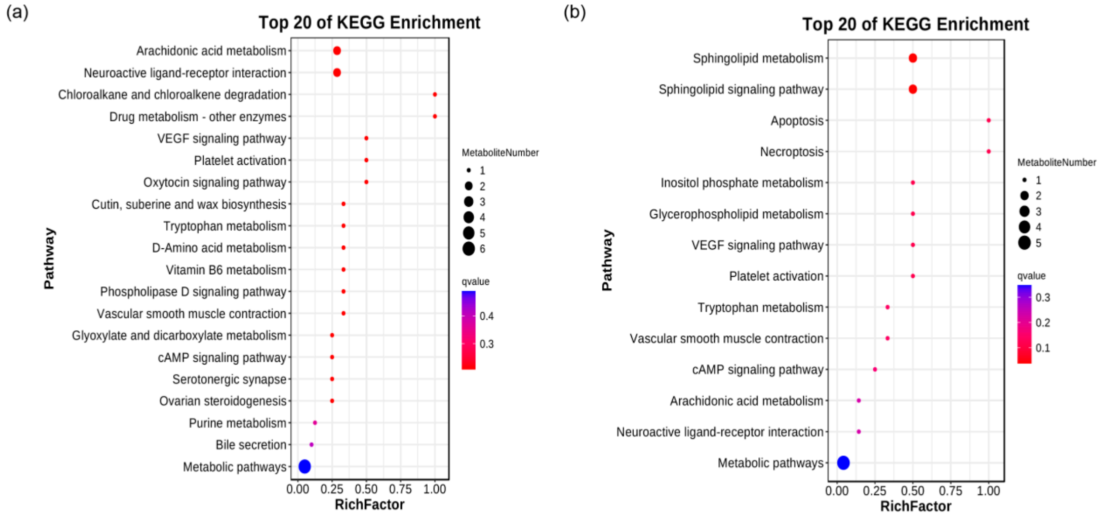

Figure S4. Bubble map illustrates KEGG pathway enrichment analysis of differential metabolites in BS_vs_RS (a) and BS_vs_RSSI (b), respectively.
